# Isolated Dupuytren’s Disease in Proximal Phalanx of the Little Finger Mimicking Giant-Cell Tumor: A Rare Case Presentation

**DOI:** 10.3390/reports9030253

**Published:** 2026-08-04

**Authors:** Grigorios Kastanis, Mikela-Rafaella Siligardou, Nikolaos Ritzakis, Alexandros Tsioupros, Constantinos Chaniotakis

**Affiliations:** 1 Reconstructive Hand Surgery Unit, “Venizeleion” General Hospital of Heraklion, 71409 Heraklion, Crete, Greece; 2 Department of Orthopaedics and Traumatology, “Venizeleion” General Hospital of Heraklion, 44 Leoforos Knossou, 71409 Heraklion, Crete, Greece; mickela_sr@yahoo.gr (M.-R.S.); nikosritz7@gmail.com (N.R.); alexandros-tsioupros@hotmail.com (A.T.)

**Keywords:** digital deformity, interphalangeal joint, Dupuytren’s disease, histological examination, contracture

## Abstract

**Background and Clinical Significance**: Dupuytren’s disease (DD) is characterized by abnormal myofibroblast proliferation and excessive collagen deposition, leading to the formation of pathological fibrous cords. It typically affects the palmar surface of the hand, where these contractile cords cause progressive flexion contractures of the metacarpophalangeal (MCP) and proximal interphalangeal (PIP) joints. Lesions involving the proximal interphalangeal (PIP) joint without significant flexion contracture may be misdiagnosed as soft-tissue tumors or inflammatory lesions based on imaging findings, including magnetic resonance imaging (MRI) and ultrasound; **Case Presentation**: We present a case of a soft-tissue mass located on the volar aspect of the proximal phalanx of the little finger, associated with a mild PIP joint contracture. The initial MRI findings suggested a giant-cell tumor of the tendon sheath; however, the diagnosis of Dupuytren’s disease was established only after histopathological examination; **Conclusions**: This case highlights the importance of considering DD in the differential diagnosis of peripheral soft-tissue lesions of the finger, particularly when presenting with only mild PIP joint contracture and atypical imaging features.

## 1. Introduction and Clinical Significance

Dupuytren’s disease (DD) is characterized by abnormal myofibroblast proliferation and excessive collagen deposition, leading to the formation of pathological fibrous cords. These cords arise from the pretendinous bands of the palmar fascia and frequently extend distally in continuity with the digital cords [1]. The disease typically manifests as progressive shortening and thickening of the palmar fascia, resulting in flexion contractures of the fingers, most commonly affecting the ring and little fingers [2].

Isolated Dupuytren’s contracture involving the proximal interphalangeal (PIP) joint is uncommon and has been reported only in isolated case reports, accounting for approximately 5% of cases [3]. Unlike the classic presentation, which primarily affects the pretendinous cords of the palm and metacarpophalangeal (MCP) joints, isolated disease at the level of the proximal phalanx or PIP joint often lacks the characteristic palmar nodules and cords, making the diagnosis considerably more challenging [4,5,6,7,8,9]. The atypical location of the lesion may lead clinicians to suspect alternative pathological entities. The differential diagnosis includes benign soft-tissue tumors, such as giant-cell tumor of the tendon sheath, fibroma of the tendon sheath, fibromatosis, ganglion cyst, and peripheral nerve sheath tumors, as well as inflammatory or infectious conditions, including localized tenosynovitis, rheumatoid nodules, gouty tophi, and chronic granulomatous processes [1,4]. Less frequently, malignant soft-tissue neoplasms, such as synovial sarcoma or fibrosarcoma, should also be considered, particularly in cases demonstrating rapid enlargement, persistent pain, or aggressive imaging characteristics [5,6,10].

Imaging studies may further complicate the diagnostic process. Magnetic resonance imaging (MRI) typically demonstrates a poorly circumscribed lesion with low-to-intermediate signal intensity on T1-weighted images and variable signal intensity on T2-weighted sequences, depending on the relative proportions of collagen and cellularity. These findings are nonspecific and may overlap with those of benign fibrous lesions, tenosynovitis, giant-cell tumor of the tendon sheath, or even low-grade soft-tissue sarcomas [11].

The diagnosis of isolated PIP joint DD requires a high index of clinical suspicion and careful correlation between the patient’s clinical presentation, physical examination, imaging findings, and intraoperative observations [3]. Histopathological examination remains the gold standard for establishing the diagnosis, demonstrating the characteristic proliferation of spindle-shaped myofibroblasts embedded within abundant collagenous stroma [3,6,9]. Recognition of this rare presentation is essential to avoid misdiagnosis, unnecessary extensive investigations, or overly aggressive surgical treatment.

We present the case of a woman with a soft-tissue mass on the volar aspect of the proximal phalanx of the little finger. The initial MRI findings were suggestive of a giant-cell tumor of the tendon sheath. However, following surgical excision, histopathological examination confirmed the diagnosis of DD. This case highlights the importance of considering DD in the differential diagnosis of isolated soft-tissue masses of the proximal phalanx, particularly when associated with a mild PIP joint contracture.

## 2. Case Presentation

A 46-year-old woman was referred from another medical center with a slowly enlarging mass on the volar aspect of the proximal phalanx of the left little finger. The patient reported that the lesion had gradually increased in size over the previous six months and denied any history of trauma. Her medical history was unremarkable and revealed no known risk factors associated with DD. Physical examination demonstrated a 2.0 × 1.5 cm, round, non-tender soft-tissue mass on the volar aspect of the proximal phalanx of the little finger. The lesion was soft in consistency, and the overlying skin appeared normal. Neurovascular examination was intact, with no sensory or motor deficits. A mild flexion contracture of the PIP joint was also present. There were no clinical signs of infection, and laboratory inflammatory markers were within normal limits.

**Figure 1:**
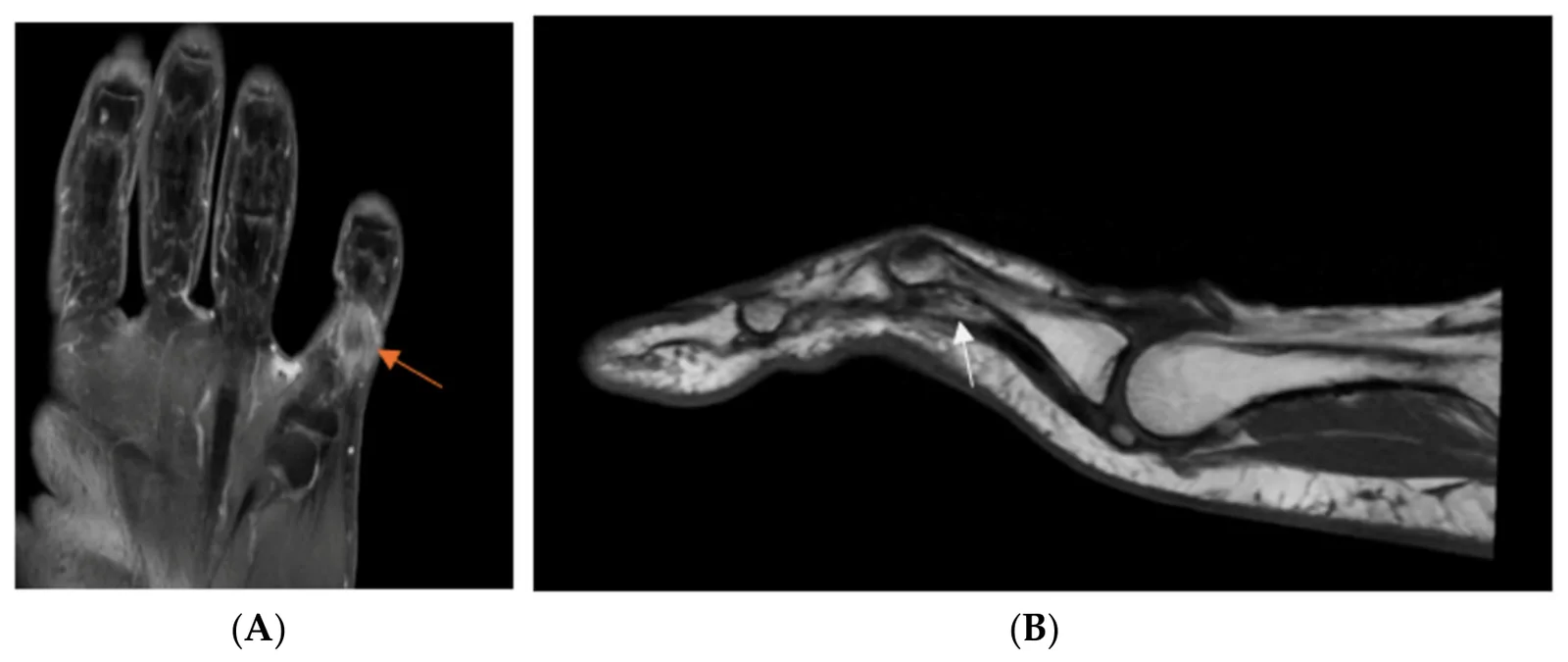
MRI ((**A**): T2—view and (**B**): T1—view) shows a solid nodular lesion (orange arrow) on the palmar surface of the proximal phalangeal of the little finger, which comes into direct contact with the flexor tendon sheath, mimicking Giant-Cell Tumor of the tendon sheath (white arrow).

MRI of the hand demonstrated a solid nodular lesion on the volar aspect of the proximal phalanx of the little finger, in direct contact with the flexor tendon sheath. The lesion was located within the subcutaneous tissue, and the radiological diagnosis was consistent with a giant-cell tumor of the tendon sheath (Figure 1). As the patient wished to have the lesion removed, an excisional biopsy was planned.

**Figure 2:**
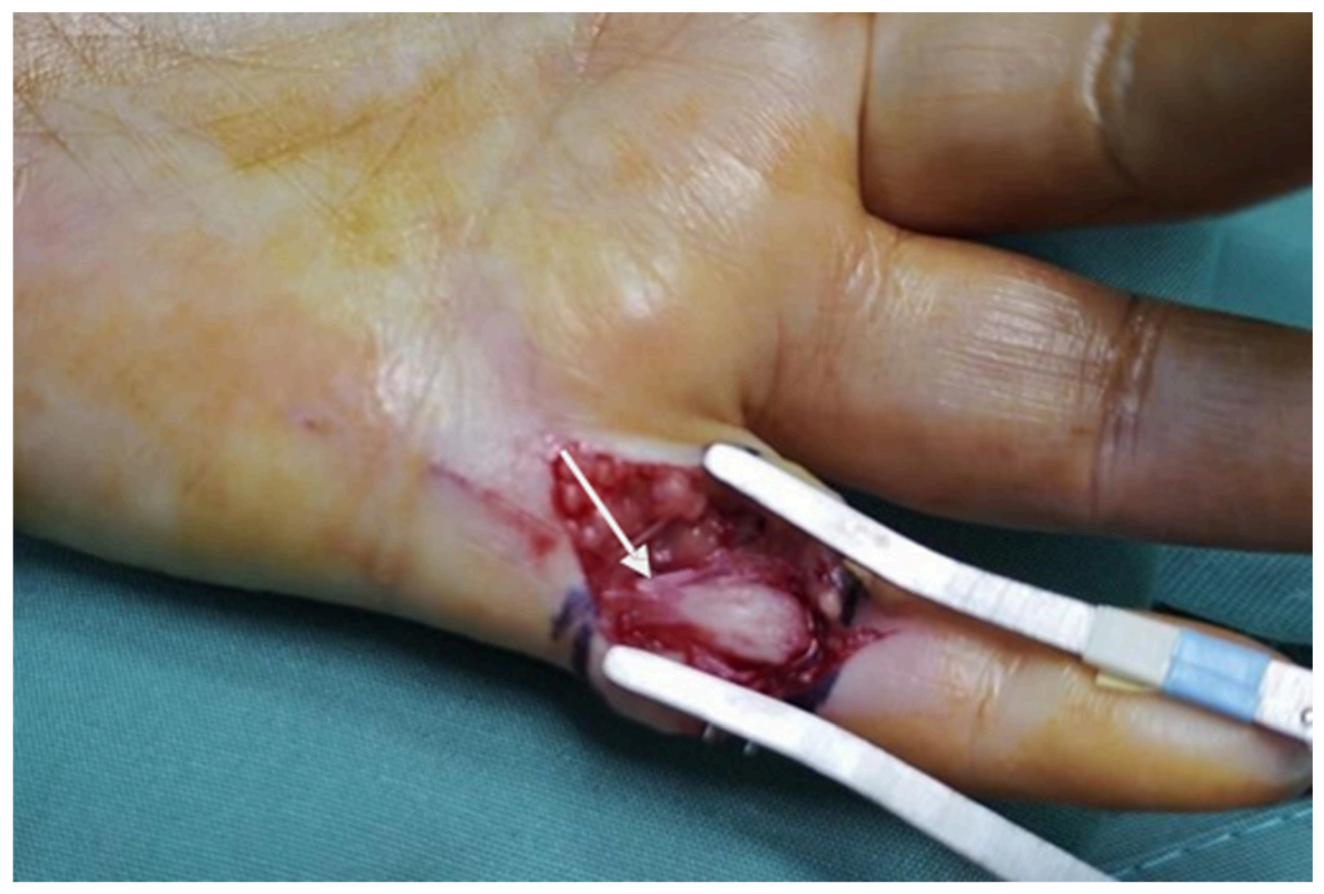
Intraop Intraoperative views show Dupuytren cord (white arrow) in the proximal digit of the little finger.

The procedure was performed under wide-awake local anesthesia with no tourniquet (WALANT). A mid-lateral incision was made over the proximal phalanx. Intraoperatively, a Dupuytren cord was identified, extending from the metacarpophalangeal (MCP) joint to the PIP joint (Figure 2). The cord overlaid with the flexor tendons, pulley system, and the ulnar neurovascular bundle of the little finger. It was carefully dissected and completely excised, with meticulous preservation of the adjacent ulnar neurovascular bundle. Intraoperatively, the lesion was carefully evaluated, and the surgical findings were not suggestive of a giant-cell tumor of the tendon sheath. Based on the macroscopic appearance and the involvement of the surrounding tissues, a classical fasciectomy was performed. The wound was closed with interrupted sutures and dressed with a sterile dressing and a volar extension plaster splint.

**Figure 3:**
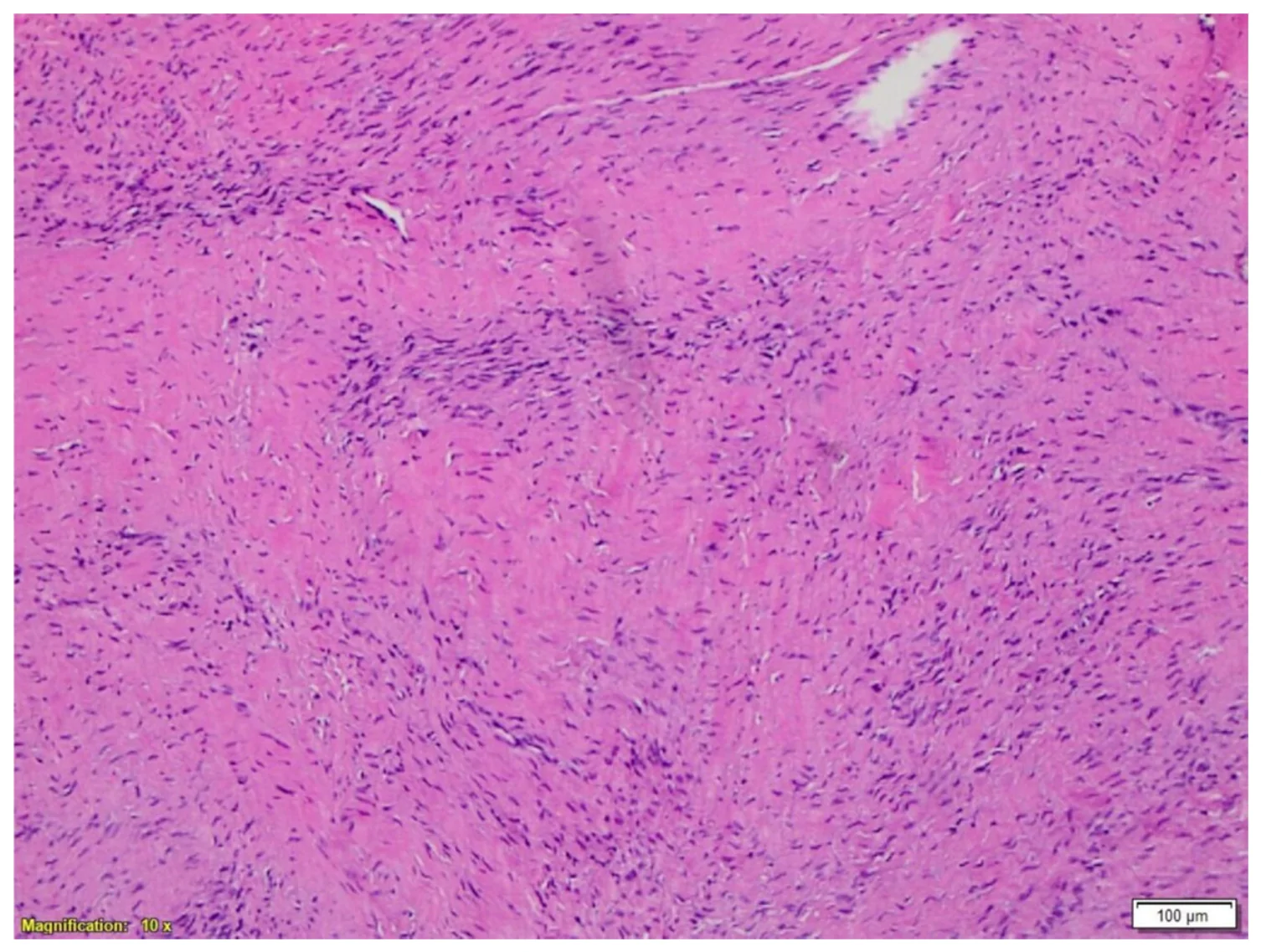
This histologic image shows a fibroblastic lesion composed of dense, hyalinized bundles with scattered spindled-shaped fibroblasts, with minimal cytologic atypia, and slit-like vascular spaces. Mitotic figures are absent. These features are consistent with DD.

The excised specimen was submitted for histopathological examination (Figure 3), which confirmed the diagnosis of DD.

**Figure 4:**
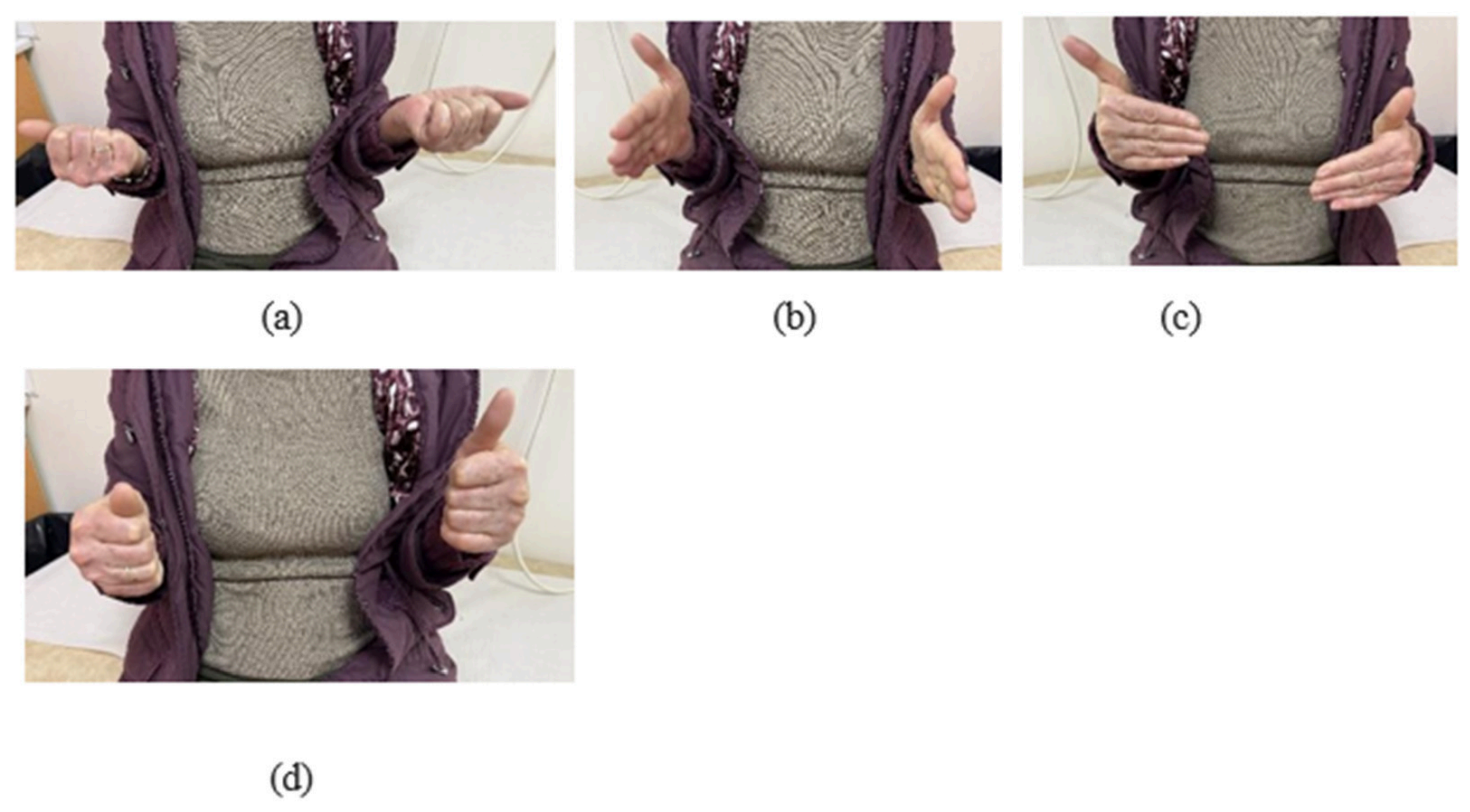
Range of motion without any indication of recurrence during six months of follow-up. As demonstrated in the clinical figures (**a**–**d**), the range of motion is identical in the affected and the contralateral healthy hand, with no observable difference between the two sides.

The postoperative course was uneventful, with no complications. At six months of follow-up, the patient demonstrated an excellent functional outcome with no clinical evidence of recurrence (Figure 4).

## 3. Discussion

DD is a progressive fibroproliferative disorder characterized by abnormal myofibroblast proliferation and excessive collagen deposition within the palmar fascia, resulting in the formation of pathological fibrous cords with variable clinical severity [1,4]. These cords originate from the pretendinous bands of the palmar fascia and progressively extend distally in continuity with the digital cords. As the disease progresses, shortening and thickening of the palmar fascia lead to progressive flexion contractures of the fingers.

The disease is commonly bilateral and may be associated with other fibroproliferative disorders, including knuckle pads (Garrod’s pads), plantar fibromatosis (Ledderhose disease), and penile fibromatosis (Peyronie’s disease) [5]. Although the pathogenesis of DD remains incompletely understood, several risk factors have been implicated, including smoking, excessive alcohol consumption, diabetes mellitus, epilepsy, carpal tunnel syndrome, frozen shoulder, rheumatoid arthritis, manual labor, repetitive hand trauma, and genetic predisposition [3]. DD occurs predominantly in men, with a male-to-female ratio of approximately 6:1, and most frequently affects the ring and little fingers [5].

According to the literature, sporadic cases have been presented in which the joint contractures included the isolated PIP joint [1,6,7,8]. Based on these findings, we hypothesize that our case represents a rare case of digital DD, in which myofibroblast proliferation occurred within the proximal phalanx independently of the MCP joint and without involvement of the palmar fascia. This atypical anatomical presentation underscores the uniqueness of the case, as the lesion clinically and radiologically mimicked a soft tissue tumor, leading to an initial misdiagnosis.

The unusual location of the mass and the absence of predisposing factors for DD prompted the use of MRI to further characterize the lesion and differentiate between a neoplastic and inflammatory process. The incorrect MRI-based diagnosis in our case highlights the importance of thorough clinical evaluation and maintaining a high index of suspicion for DD when encountering atypical digital lesions. Ultimately, histopathological examination remains essential for establishing the definitive diagnosis.

Treatment of DD is determined according to the stage of the disease and patient-specific factors. Conservative treatment options include padded fingerless gloves, radiotherapy, and collagenase injections, whereas surgical management involves resection of the affected palmar aponeurosis, a procedure known as fasciectomy [2]. Ruettermann et al. proposed a treatment algorithm aimed at guiding the therapeutic management of DD [9]. According to this approach, painful fibrotic lesions without functional extension deficits can usually be managed conservatively, while surgical intervention should be considered only when contractures develop that significantly impair hand function and affect the patient’s quality of life [9]. In our case, surgical excision was performed based on the initial diagnosis of a giant-cell tumor of the tendon sheath. However, intraoperatively, the characteristic fibrous cords associated with DD were identified. Therefore, complete excision of the lesion was performed in combination with fasciectomy.

The differential diagnosis of DD can be challenging, particularly in atypical presentations involving isolated digital lesions without the classic palmar involvement or significant contracture. In these cases, DD may mimic other benign soft-tissue lesions, including giant-cell tumor of the tendon sheath, fibroma of the tendon sheath, ganglion cysts, and other fibroproliferative or inflammatory lesions [10,11]. Although magnetic resonance imaging (MRI) and ultrasound can provide valuable information regarding lesion size, morphology, and relationship with adjacent anatomical structures, imaging findings may overlap with those of other soft tissue pathologies, resulting in potential diagnostic errors, especially in atypical presentations of DD [10]. On MRI, giant-cell tumor of the tendon sheath generally appears as a well-defined lesion with low signal intensity on both T1- and T2-weighted sequences due to abundant hemosiderin deposition, often demonstrating blooming artifacts on gradient-echo images. In contrast, DD usually presents as an ill-defined fibrous proliferation arising from the superficial palmar fascia or digital fascia, with signal characteristics that vary according to the degree of collagen deposition and cellularity [11]. Nevertheless, these imaging features may overlap, particularly in atypical digital manifestations of DD, making radiological distinction difficult [11].

Definitive differentiation relies on histopathological examination, with DD demonstrating proliferating spindle-shaped myofibroblasts embedded in dense collagenous stroma, whereas giant-cell tumor of the tendon sheath is characterized by a mixture of mononuclear histiocyte-like cells, osteoclast-like multinucleated giant cells, foam cells, and hemosiderin-laden macrophages [9,10,11].

Therefore, a high index of clinical suspicion, careful physical examination, and histopathological evaluation remain essential for establishing the definitive diagnosis of DD in unusual anatomical locations [10,12].

## 4. Conclusions

DD is characterized by progressive idiopathic fibrosis of the palmar aponeurosis, resulting in impaired hand function and the formation of fibrotic nodules and cords. The isolated localization of DD in the proximal phalanx represents a diagnostic challenge, particularly in the absence of predisposing factors or significant flexion contractures of the digits. In such cases, careful clinical examination and a high index of suspicion for atypical digital lesions are essential, while histopathological examination remains the key factor for establishing the definitive diagnosis.

## Data Availability

The original data presented in the study are included in the article, further inquiries can be directed to the corresponding author.

## Abbreviations

The following abbreviations are used in this manuscript:

MRI	Magnetic resonance imaging
DD	Dupuytren’s disease
CCH	Collagenase clostridium histolyticum
WALANT	Wide-Awake Local Anesthesia No Tourniquet
PIP	Proximal interphalangeal
DIP	Distal interphalangeal
